# Supplementation with Korean Red Ginseng Improves Current Perception Threshold in Korean Type 2 Diabetes Patients: A Randomized, Double-Blind, Placebo-Controlled Trial

**DOI:** 10.1155/2020/5295328

**Published:** 2020-01-16

**Authors:** Kahui Park, YuSik Kim, Junghye Kim, Shinae Kang, Jong Suk Park, Chul Woo Ahn, Ji Sun Nam

**Affiliations:** ^1^Division of Endocrinology, Department of Internal Medicine, Gangnam Severance Hospital, Yonsei University College of Medicine, Republic of Korea; ^2^Severance Institute for Vascular and Metabolic Research, Yonsei University College of Medicine, Republic of Korea

## Abstract

**Background:**

Many Type 2 diabetes (T2DM) patients in Korea take Korean Red Ginseng (KRG) for various reasons. In this study, we investigated the effects of KRG administration on diabetic peripheral neuropathy in T2DM patients.

**Methods:**

This study was a randomized, double-blind, placebo-controlled trial. Participants were randomly allocated to either the placebo or KRG group and took corresponding tablets for 24 weeks. The primary outcomes were changes in current perception threshold (CPT) at week 24. Secondary outcomes were altered fasting plasma glucose, HbA1c, and various metabolic and inflammatory markers at week 24.

**Results:**

Sixty-one patients completed the study. The CPT of the lower extremities at various frequencies exhibited significant improvements at week 24 in the KRG group. Other metabolic parameters were not altered after 24 weeks in both groups. In the subgroup analysis, CPT levels were improved in those with a longer diabetes duration or who already had neuropathy at the beginning of the study, and insulin resistance was improved in patients with a shorter diabetes duration.

**Conclusion:**

Twenty-four week administration of KRG in T2DM patients resulted in a significant improvement in neuropathy, especially in those with a longer diabetes duration. A further, larger population study with a longer follow-up period is warranted to verify the effects of KRG on diabetic neuropathy.

## 1. Introduction

Type 2 diabetes is the fastest-growing metabolic disease in the world, which involves various organs [1]. Chronic hyperglycemia leads to a damage in small blood vessels in peripheral nerves, kidney, and retina as well as in larger blood vessels of the heart and the brain. These chronic diabetic microvascular and macrovascular complications lead to significant mortality and morbidity in diabetes patients. Among them, diabetic neuropathy is the most common microvascular complication affecting as many as 50% of diabetes patients, and it is the leading cause of nontraumatic limb amputation [2–4].

Measurement of current perception threshold (CPT) using the Neurometer® has been proven to be a reliable method to assess diabetic neuropathy by quantifying sensory fiber function. The CPT test is widely used in clinical practice for being noninvasive and being able to detect not only advanced severe neuropathy but also early asymptomatic neuropathy compared to other sensory tests [5–7]. Despite many experimental and clinical studies, there is no effective treatment that directly affects the pathogenesis of diabetic neuropathy. We use *α*-lipoic acid to reduce oxygen free radicals, and anticonvulsants such as gabapentin and pregabalin for pain relief in painful neuropathy, but their effectiveness and persistence are limited [2, 8, 9].

Ginseng (*Panax ginseng* Meyer) roots have been widely used as an herbal treatment in East Asia for more than 2000 years. Korean Red Ginseng (KRG) is a popular traditional herb that is known to have beneficial effects in obesity, postmenopausal symptoms, cholesterol levels, and cardiovascular diseases. There are several animal and human studies to support these effects [10, 11]. Many Type 2 diabetes patients also take KRG as a complementary and alternative medicine for better glycemic control and health promotion [12]. Various animal and clinical studies have been conducted, and KRG has been shown to improve postprandial glucose and insulin sensitivity in several studies [13, 14].

Such effects of KRG are thought to be due to anti-inflammatory and antioxidant activities [15–17]. Chronic hyperglycemia with consequent oxidative stress, advanced glycation end product (AGE), and various inflammatory responses are known to mediate diabetic complications. Several animal studies have demonstrated that Red Ginseng can reduce diabetic microvascular complications and brain neuronal damage by reducing oxidative stress and advanced glycation end products (AGE) [18–20].

However, human studies on the effects of Red Ginseng on diabetic complications are lacking and the follow-up period is short. In this study, we investigated the effects of KRG administration on diabetic neuropathy in Type 2 diabetes patients.

## 2. Research Design and Methods

### 2.1. Subjects

A total of 70 Type 2 diabetes patients who visited the Gangnam Severance Hospital between April 2016 and July 2017 were enrolled in this single-center, double-blind, randomized, placebo controlled trial. Eligible patients were men and women between the ages of 19 and 75 who were diagnosed with Type 2 diabetes more than 6 months prior and those taking oral antidiabetic agents at the time of enrollment with an unchanged dose or type of drug within the last 3 months. Patients with HbA1c > 10%, eGFR < 30 ml/min/1.73 m^2^, AST/ALT > 3 times greater the upper normal limit, taking glucocorticoids or any herbal medicine within the past 3 months, chronic inflammatory disease in the active phase or acute infection status, and pregnant or lactating were excluded. Enrolled subjects were randomized using a computer-generated randomization table.

### 2.2. Design

**Figure 1:**
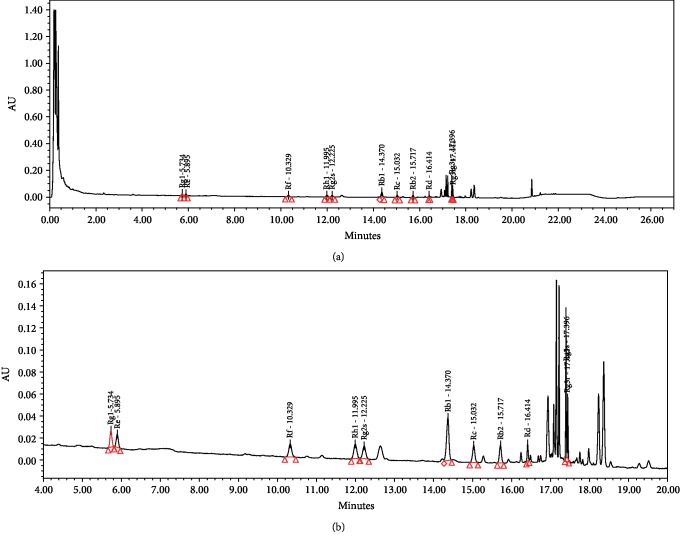
HPLC picture of KRG ginsenosides. An expanded version of (a) is shown in (b).

Subjects were randomized to receive KRG extract tablets or placebos for 24 weeks. They were instructed to take 2 KRG extract tablets, with each tablet containing 500 milligrams of KRG extract powder, which is equivalent to taking 3 g of KRG extract per day. The components of KRG are as follows: 0.37 mg/g of ginsenoside Rg1, 0.49 mg/g of ginsenoside Re, 1.23 mg/g of ginsenoside Rf, 2.35 mg/g of ginsenoside Rh1, 3.06 mg/g of ginsenoside Rg2s, 4.43 mg/g of ginsenoside Rb1, 2.01 mg/g of ginsenoside Rc, 1.71 mg/g of ginsenoside Rb2, 0.86 mg/g of ginsenoside Rd, 3.27 mg/g of ginsenoside Rg3s, 1.02 mg/g of ginsenoside Rg3r, and 88.5 mg/g of acid polysaccharide. The HPLC picture of KRG components is shown in Figure 1. The placebo group took 2 tablets that were made from the same corn starch and cellulose as the KRG tablets in the same shape and size with the addition of Red Ginseng flavor twice a day. The KRG and placebo tablets were manufactured and supplied by the Korea Ginseng Corporation (Seoul, Korea). No other medication was prescribed, and the patients' original diabetes medication remained unchanged. All subjects, clinical investigators, and outcome assessors were blind to treatment allocation.

All participants provided informed consent, and the study was approved by the Institutional Review Board of Gangnam Severance Hospital. The study was registered on ISRCTN17039603.

#### 2.2.1. Anthropometric Parameters and Biochemical Profiles

Body weight and height were measured in the morning with participants wearing light clothing. Body mass index (BMI) was calculated as body weight in kilograms divided by height in meters squared (kg/m^2^).

Blood samples were taken from the antecubital vein after at least 8 hours of fasting at weeks 0, 12, and 24. The fasting plasma glucose concentrations were measured by a standard glucose oxidase method (747 Automatic Analyzer, Hitachi, Tokyo, Japan). Fasting serum insulin was determined by chemiluminescence (RIA Kit, Daiichi, Japan), and HbA1c was measured by high-performance liquid chromatography (VARIANT II, Bio-Rad, Hercules, CA, USA). A standard 75 g oral glucose tolerance test (OGTT) was also performed, and plasma glucose and insulin levels were measured in venous blood collected at 0, 60, and 120 minutes after ingestion. Total cholesterol, high-density lipoprotein (HDL) cholesterol, low-density lipoprotein (LDL) cholesterol, and triglycerides were measured enzymatically using a chemical analyzer (Daiichi, Hitachi 747, Japan). Apolipoprotein A-1 and apolipoprotein B were measured through an immunoturbidimetric method (Cobas Integra 800 Analyzer; Roche Diagnostics) using standard procedures.

Serum blood urea nitrogen (BUN) and creatinine (Cr) levels were measured enzymatically using a chemical analyzer (AU5800, Beckman Coulter, Inc., Brea, CA, USA). Serum AST and ALT were measured for the liver function test (AU5800, Beckman Coulter, Inc., Brea, CA, USA).

#### 2.2.2. Insulin Resistance

Insulin resistance was assessed by the HOMA equation as follows: Homeostasis Model Assessment of Insulin Resistance (HOMA − IR) = [fasting glucose (mg/dl) × fasting insulin (mcIU/ml)/405].

#### 2.2.3. Diabetic Neuropathy Assessment

Diabetic peripheral neuropathy was assessed using the current perception thresholds (CPT) test by Neurometer® (Neurotron, Inc., Baltimore, MD, USA). It was measured at the beginning of the study (baseline) and after 24 weeks of medication.

The patient was to sit in a comfortable position with two electrodes coated with conductive gel attached to unilateral dorsal surfaces of the distal phalanges of the index finger and great toe. The current was increased (to a maximum of 9.99 mA) until the patient began to feel the current at the skin where the electrodes are attached. The current was then terminated, decreased by 0.08 mA, and reapplied. The patient was tested with three different alternating frequencies of electrical stimulus (2000, 250, and 5 Hz). At each frequency, the current was slowly increased until the subject first reported the perception of sensation. The current was then decreased and reincreased until a consistent threshold is measured [6].

#### 2.2.4. Complication-Related Mediators

The serum levels of 8-epi-PGF2*α* (Cloud-Clone Corp., CEA749Ge), high-sensitivity CRP (CusaBio, CSB-E08617h), TNF-*α* (Cloud-Clone Corp., SEA133Hu), and AGEs (Cloud-Clone Corp., CEB353Ge) were quantified by enzyme-linked immunosorbent assay. Blood samples were centrifuged immediately (1600 g, 10 minutes), and the serum was stored at -70°C until analysis. All procedures were performed according to the manufacturer's instructions.

### 2.3. Statistical Analysis

The primary outcomes were changes of CPT levels at week 24. Secondary outcomes were changes in fasting plasma glucose, HbA1c, and various metabolic and inflammatory mediators of diabetic complications at weeks 0 and 24.

Statistical analysis was performed using SPSS version 23.0 for Windows. All data were presented as mean ± SD. The independent *t*-test was used to compare changes from baseline to 24 weeks between the two groups. The paired *t*-test was used to compare baseline data with changes at 24 weeks for each group. A value of *p* < 0.05 was considered statistically significant.

## 3. Results

### 3.1. Patient Characteristics

**Figure 2:**
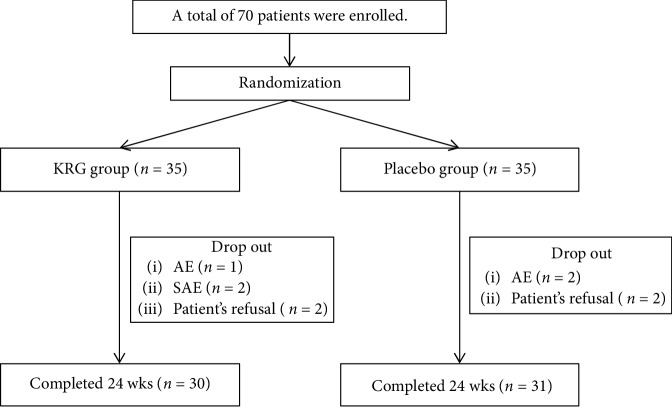
Trial flow.

During the study period, 9 patients dropped out due to a lack of time, lack of compliance, and adverse effects. A total of 61 patients (30 patients in the KRG group and 31 in the placebo group) completed the study (Figure 2). The patients' baseline characteristics are outlined in Table 1. The participants' mean age was 59 years, mean BMI was 25 kg/m^2^, and mean diabetes duration was 12 years. Approximately one-third of the patients were taking one type of oral hypoglycemic agent and two-thirds were taking more than two agents. There were no significant differences between the two groups in terms of smoking and alcohol consumption.

### 3.2. Changes in Metabolic Parameters

There were no differences in BMI and blood pressure after 24 weeks of medication. Also, there were no significant differences regarding HbA1c, fasting plasma glucose, and insulin levels in both groups. As an index of insulin resistance, HOMA-IR was elevated after 24 weeks in both the KRG and placebo groups, but was not statistically significant. Lipid profiles (total cholesterol, triglyceride, apolipoprotein A1, apolipoprotein B, HDL, and LDL-cholesterol) also exhibited no statistically significant changes after 24 weeks of drug administration (Table 2).

### 3.3. Changes in CPT and Diabetic Complication-Related Mediators

CPT was significantly improved in both the left and right lower extremities at various frequencies in the KRG group, while most of the CPT did not show statistically significant improvement in the placebo group (Table 3).

8-epi-PGF2*α*, advanced glycation end product (AGE), and inflammatory markers (hsCRP, IL-6, and TNF-*α*) were not significantly altered in both the KRG and placebo groups (Table 3).

### 3.4. Subgroup Analysis

Subjects were divided into subgroups according to the duration of diabetes and the presence of diabetic microvascular complications.

In subjects with diabetes duration of less than 5 years, fasting insulin decreased from 11.1 ± 4.1 to 6.3 ± 2.1 mcIU/ml, and HOMA-IR improved significantly from 3.39 ± 1.2 to 2.08 ± 0.8 after KRG treatment (*p* = 0.044 and *p* = 0.046, respectively). In the patients with diabetes duration of more than 5 years, the CPT of lower extremities were significantly improved after KRG administration from 299.42 ± 81 to 258.92 ± 86 at 2000 Hz (*p* = 0.012) and from 137.13 ± 43 to 106.96 ± 36 at 250 Hz (*p* = 0.002), while there were no significant changes in the placebo group.

In patients who already had abnormal CPT at baseline, the CPT levels at 2000 Hz decreased significantly from 323.24 ± 91 to 278.94 ± 93 (*p* = 0.049), and the CPT levels at 250 Hz decreased significantly from 145.65 ± 46 to 119.35 ± 36 (*p* = 0.028) after KRG administration. These changes were not observed in the placebo group.

### 3.5. Safety

There were no serious adverse effects in both the KRG and placebo groups. Three patients in the KRG group dropped out due to fever and gastrointestinal disturbance, foot pain, and deterioration of blood glucose. In the placebo group, two patients dropped out due to fever, palpitations, and fatigue. Serum AST and ALT levels did not deteriorate, and hypoglycemia did not occur during the study period in both groups.

## 4. Discussion

In this study, we investigated the effects of KRG on diabetic neuropathy in Type 2 diabetes patients. KRG is a Korean traditional herb that is becoming popular worldwide for its beneficial effects in obesity, insulin resistance, dyslipidemia, and cardiovascular diseases due to its antioxidant and anti-inflammatory effects. Many diabetes patients take KRG for the above reasons. However, there has been no study that investigated its effect on diabetic neuropathy, which is the most common diabetic microvascular complication that does not have a definite pathogenic treatment option.

Twenty-four weeks of KRG administration resulted in improved peripheral neuropathy particularly in those with a longer diabetes duration and underlying neuropathy. Meanwhile, insulin sensitivity improved in those with a relatively short diabetes duration. We believe our study results have significance for its possible beneficial effects in diabetic neuropathy without serious adverse events.

Diabetic neuropathy is the most common diabetic chronic complication that results in pain, disability, and amputation. Its pathogenesis is not fully understood yet, but toxic effects of chronic hyperglycemia involving oxidative stress, polyol pathway, and advanced glycation end products (AGE) are known to play important roles [21–25]. Ginseng has been shown to have antioxidant and anti-inflammatory properties, and thus studies have been conducted to assess the effects of ginseng on diabetic microvascular complications including neuropathy, retinopathy, and nephropathy in diabetic animal models [26, 27]. However, there has been no human study so far.

In this study, the CPT test, which is a quick, convenient, but also a reliable way to assess diabetic neuropathy was used [28]. The KRG group showed consistent and statistically significant improvements of CPT levels throughout different frequencies of stimulus. Meanwhile, the placebo group also showed improved CPT, but mostly it did not reach statistical significance. In particular, CPT levels at higher frequencies were significantly improved in the KRG group. The CPT levels at 2000 or 250 Hz are more clinically important than those at 5 Hz, because it has been shown that the Neurometer® displays poor reproducibility at low frequencies like 5 Hz compared to 250 and 2000 Hz [29]. Also, it was notable that CPT was significantly improved in patients with longer diabetes duration and in those who already had an impaired CPT at baseline.

We have assessed various factors known to mediate chronic diabetic complications to investigate possible mechanisms for such improvement. We investigated AGE and various oxidative and inflammatory markers such as high-sensitivity C-reactive protein, interleukin 6, TNF-*α*, and 8-epi-PGF2*α*. However, they were not altered after 24 weeks of KRG and did not show significant correlations with changes in CPT. There have been many studies assessing the effects of natural compounds with antioxidant or anti-inflammatory effects on diabetic complications, and many showed benefits. For example, an animal study conducted on streptozotocin-induced diabetes mice demonstrated a decreased AGE level in urine and kidney as well as plasma TNF-*α* and reduced renal damage after KRG administration [16].

However, like our study, most of the human studies conducted with natural compounds on diabetic neuropathy failed to elucidate the underlying mechanism [26]. First of all, compared to diabetic nephropathy, it is harder to assess diabetic neuropathy and its mechanism is more complex. Also, in contrast to pharmacologically manufactured products with high potency and predictable pharmacodynamics, natural compounds like KRG tend to have diverse pleiotropic effects rather than one strong specific effect. Therefore, although their properties can be observed in in vivo experiments, it may be too moderate to be seen at the human blood level. In other words, the effect of KRG on neuropathy may have resulted not from one strong mechanism but rather from multiple mechanisms that interact with each other.

There have been inconsistent data for the effects of KRG on blood glucose control, insulin resistance, and various metabolic parameters. In this study, the administration of KRG for 24 weeks did not affect patient BMI, blood pressure, and blood glucose. Regarding insulin resistance, changes in fasting insulin and HOMA-IR were not significantly different between the two groups. However, in the subgroup analysis with patients whose duration of diabetes mellitus was relatively short (less than 5 years), fasting insulin significantly decreased from 11.1 to 6.3 mcIU/ml and HOMA-IR significantly improved from 3.39 to 2.08 (*p* = 0.044 and *p* = 0.046, respectively). These results are in line with previous studies that KRG improves peripheral insulin resistance [10, 14, 30, 31].

In terms of safety, there was no hepatotoxicity, nephrotoxicity, or hypoglycemia in the KRG group. Among the 3 patients in the KRG group who dropped out due to adverse effects, one was admitted to the hospital due to deterioration in blood glucose level. However, this was because the patient did not properly take the prescribed antidiabetic drugs and had an uncontrolled diet. Another patient in the KRG group complained of fever and gastrointestinal disturbance, but there was one patient with similar symptoms in the placebo group. The third patient complained of foot pain, but there was no sign of diabetic foot. In the placebo group, one patient dropped out due to fever and fatigue, while another patient exhibited elevated liver enzymes.

There are several limitations to this study. First of all, the number of study subjects was small, and the study duration was relatively short to assess chronic diabetic complications. However, this was the first randomized controlled trial to assess the effects of KRG on diabetic neuropathy in Type 2 diabetes patients. Secondly, the underlying mechanism for improved CPT and insulin resistance following KRG administration had not been elucidated. Thirdly, there is no data after the discontinuation of KRG to examine the reversal effect. Also, all the subjects took the same dose of KRG, which is the commercially available dose in Korea, regardless of their body weight. This study would have observed more precise KRG effects if the dose was titrated to body weight. Lastly, subjects with renal or hepatic dysfunction were excluded from the study, and therefore, the effects and safety of KRG cannot be generalized.

In conclusion, in this first randomized controlled trial to assess the effects of KRG on chronic diabetic neuropathy, a 24-week administration of KRG significantly improved markers of diabetic neuropathy especially in those who already had neuropathy or a long diabetes duration. In addition, it also improved insulin resistance in patients with a short diabetes duration. Considering that diabetic microvascular complication is a chronic progressive disease, a further, larger population study with a longer follow-up period is warranted to verify and understand the effects of KRG on diabetic complications.

## Figures and Tables

**Table 1:** Subject characteristics at baseline.

	KRG group (*n* = 30)	Placebo group (*n* = 31)	*p* value^a^
Gender (M/F)	17/13	19/12	NS^a^
Age (years)	59.3 ± 8.79	59.7 ± 7.22	NS
BMI (kg/m^2^)	24.7 ± 2.93	24.8 ± 2.82	NS
DM duration (years)	11.6 ± 6.80	12.6 ± 8.83	NS
DM medication			
Monotherapy	9 (30.0%)	11 (35.5%)	
Dual therapy	13 (43.3%)	8 (25.8%)	NS^b^
Triple therapy	18 (26.7%)	12 (38.7%)	
SBP (mmHg)	131.6 ± 15.64	132.2 ± 16.77	NS

DBP (mmHg)	77.7 ± 8.71	80.8 ± 12.03	NS
Smoking			
Nonsmoker	16 (53.3%)	20 (64.5%)	
Ex-smoker	3 (10.0%)	5 (16.1%)	NS^b^
Current smoker	11 (36.7%)	6 (19.4%)	
Alcohol			
No	22 (73.3%)	22 (71.0%)	NS^b^
Yes	8 (26.7%)	9 (29.0%)	

Data are presented as mean ± SD. ^a^Analyzed by independent *t*-tests and the *p* value represents the comparison to the placebo group. ^b^Analyzed by Chi-square. KRG: Korean Red Ginseng; BMI: body mass index; SBP: systolic blood pressure; DBP: diastolic blood pressure.

**Table 2:** Anthropometric and biochemical profile.

	KRG group (*n* = 30)			Placebo group (*n* = 31)		
	Week 0	Week 24	Within-group *p* value	Week 0	Week 24	Within-group *p* value
BMI (kg/m^2^)	24.8 ± 2.96	24.5 ± 2.75	NS	24.4 ± 2.68	24.2 ± 2.76	NS
SBP (mmHg)	132 ± 15.91	133 ± 15.21	NS	132 ± 16.88	130 ± 19.18	NS
DBP (mmHg)	78 ± 8.77	80 ± 9.13	NS	80 ± 12.11	79 ± 13.84	NS
HbA1c (%)	7.4 ± 0.79	7.5 ± 0.83	NS	7.4 ± 0.70	7.7 ± 0.85	NS
FPG (mg/dl)	143 ± 30.41	150 ± 31.98	NS	153 ± 30.09	159 ± 39.33	NS
2 h Glc (mg/dl)	305 ± 65.31	307 ± 74.60	NS	301 ± 67.49	306 ± 79.25	NS
Ins (mcIU/ml)	7.6 ± 4.51	7.3 ± 4.34	NS	7.8 ± 5.08	7.8 ± 5.12	NS
2 h Ins (mcIU/ml)	31.6 ± 17.93	31.6 ± 20.03	NS	32.1 ± 23.53	29.4 ± 21.12	NS
HOMA-IR	2.6 ± 1.71	2.7 ± 1.84	NS	2.9 ± 1.80	3.1 ± 2.42	NS
TC (mg/dl)	171 ± 36.14	179 ± 41.51	NS	170 ± 33.64	180 ± 39.62	NS
TG (mg/dl)	167 ± 130.82	160 ± 93.52	NS	141 ± 60.90	182 ± 177.19	NS
HDL-C (mg/dl)	48 ± 10.44	49 ± 8.65	NS	50 ± 9.56	50 ± 11.16	NS
LDL-C (mg/dl)	114 ± 28.80	119 ± 34.27	NS	113 ± 29.35	117 ± 30.75	NS
Apo A1 (mg/dl)	139.3 ± 17.88	140.0 ± 17.66	NS	146.8 ± 20.34	145.3 ± 22.87	NS
Apo B (mg/dl)	89.6 ± 19.29	95.6 ± 25.84	NS	90.4 ± 21.49	95.2 ± 22.53	NS

BMI: body mass index; SBP: systemic blood pressure; DBP: diastolic blood pressure; FPG: fasting plasma glucose; 2 h Glc: 2-hour glucose; Ins: fasting insulin; 2 h Ins: 2-hour insulin; TC: total cholesterol; TG: triglyceride; HDL-C: HDL-cholesterol; LDL-C: LDL-cholesterol; Apo A1: apolipoprotein A1; Apo B: apolipoprotein B.

**Table 3:** Diabetic microvascular complication studies.

	KRG group (*n* = 30)			Placebo group (*n* = 31)		
	Week 0	Week 24	Within-group *p* value	Week 0	Week 24	Within-group *p* value
CPT of Lex, right						
2000 Hz	306.07 ± 82	266.79 ± 81	0.010^∗^	295.54 ± 87	256.79 ± 80	NS
250 Hz	135.64 ± 40	110.07 ± 36	0.007^∗^	140.68 ± 55	118.93 ± 35	NS
5 Hz	88.61 ± 38	76.07 ± 25	NS	93.11 ± 33	82.79 ± 33	NS
CPT of Lex, left						
2000 Hz	300.79 ± 82	267.14 ± 82	0.023^∗^	288.86 ± 93	266.86 ± 72	NS
250 Hz	130.07 ± 40	105.18 ± 35	0.005^∗^	137.29 ± 67	107.39 ± 35	0.022^∗^
5 Hz	85.54 ± 25	69.04 ± 23	0.007^∗^	91.71 ± 35	70.96 ± 27	0.004^∗^
PGF2*α* (pg/ml)	132.48 ± 109.50	125.89 ± 110.00	NS	151.34 ± 143.06	137.73 ± 122.23	NS
hsCRP (mcg/ml)	2.55 ± 7.47	0.61 ± 0.26	NS	1.56 ± 2.51	1.04 ± 1.73	NS
IL-6 (pg/ml)	2.90 ± 6.21	2.09 ± 1.75	NS	1.86 ± 1.48	1.83 ± 1.57	NS
TNF-*α* (pg/ml)	10.70 ± 20.82	14.73 ± 34.26	NS	3.11 ± 4.41	2.96 ± 4.33	NS
AGE (mcg/ml)	2.01 ± 1.06	2.11 ± 1.03	NS	1.89 ± 0.96	1.93 ± 0.94	NS

^∗^ *p* ≤ 0.05 is significant. CPT: current perception threshold; Lex: lower extremities; PGF2*α*: isoprostane 8-epi PGF2*α*; hsCRP: high-sensitivity C-reactive protein; IL-6: interleukin 6; TNF-*α*: TNF-alpha; AGE: advanced glycation end product.

## Data Availability

The data used to support the findings of this study are available from the corresponding author upon request.

## Abbreviations

AGE: Advanced glycation end products
CPT: Current perception threshold
KRG: Korean Red Ginseng.
